# Dysregulated lncRNA-UCA1 contributes to the progression of gastric cancer through regulation of the PI3K-Akt-mTOR signaling pathway

**DOI:** 10.18632/oncotarget.19281

**Published:** 2017-07-17

**Authors:** Chengyun Li, Geyu Liang, Sheng Yang, Jing Sui, Wenzhuo Yao, Xian Shen, Yanqiu Zhang, Hui Peng, Weiwei Hong, Siyi Xu, Wenjuan Wu, Yancheng Ye, Zhiyi Zhang, Wenhua Zhang, Lihong Yin, Yuepu Pu

**Affiliations:** ^1^ Key Laboratory of Environmental Medicine Engineering, Ministry of Education, School of Public Health, Southeast University, Nanjing, Jiangsu 210009, P.R. China; ^2^ Gansu Wuwei Tumor Hospital, Wuwei, Gansu 733000, P.R. China

**Keywords:** gastric cancer, lncRNA, UCA1, progression, molecular mechanism

## Abstract

The long non-coding RNA (lncRNA) urothelial carcinoma-associated 1 (UCA1) has been recently shown to be dysregulated during disease occurrence and to play an important role in the progression of several cancers. However, the biological role and potential regulation mechanism of UCA1 in the carcinogenesis of gastric cancer remain unclear. In the present study, we found that UCA1 was aberrantly upregulated in gastric cancer tissues and gastric cancer cell lines, and was associated with TNM stage and metastasis. UCA1 silencing significantly inhibited gastric cancer BGC-823 cell proliferation and increased its apoptosis. We also found that UCA1 played an important role in the migration and invasion of gastric cancer cells *in vitro* and *in vivo*. The molecular mechanism of UCA1 suggested that UCA1 regulates the PI3K-Akt-mTOR signaling proteins and their downstream mediators, to alter gastric cancer progression *in vitro* and *in vivo*. Collectively, the results showed a pivotal role of UCA1 in the tumorigenesis of gastric cancer. In addition, the study characterized a novel lncRNA-mRNA regulatory network, which may lead to a better understanding of the pathogenesis of gastric cancer and assist in lncRNA-directed diagnosis and therapy for this malignancy.

## INTRODUCTION

Gastric cancer is one of the most common cancers in the world. Every year, approximately 1 million patients are diagnosed with gastric cancer, and approximately 0.75 million patients die from this disorder [1]. Although patients during the early stages of gastric cancer can be cured by surgery, most gastric cancer patients are diagnosed at advanced stages and present with extensive invasion, lymphatic metastasis, and other organ metastases [2]. Improvements in the efficiency of early stage gastric cancer diagnosis and validation, and identification of diagnostic and prognostic biomarkers are the important research objectives. These objectives can be accomplished by investigating the pathogenesis and identification of the genetic changes, to find diagnostic markers that can be used in novel effective therapies and treatments for the prevention of gastric cancer.

The discovery of long non-coding RNA (lncRNA) in the human genome has provided a new direction in cancer research [3]. LncRNAs, which are more than 200 bases in length with little or no protein coding capacity, regulate protein expression through promotion of translation or indirect degradation of RNA transcripts in a sequence specific manner [4–6]. Recently, increasing studies have reported that lncRNAs are aberrantly expressed in many human cancers [7–9]. Thus, lncRNA functions may play important roles in the progression and metastasis of cancers. Identification of differentially expressed lncRNAs in gastric cancer, and further characterization of their functions and mechanisms is important in the diagnoses and identification of prognostic biomarkers for this disorder.

In previous studies, we used microarray analyses to screen differential expression profiles of lncRNAs from advanced gastric cancer tissues and adjacent non-tumor tissues, to show significantly higher expression of the lncRNA urothelial carcinoma-associated 1 (UCA1) [10]. Our previous studies also characterized the differential expression profiles of lncRNAs in gastric cancer using data sets and The Cancer Genome Atlas (TCGA) [11]. A total of 361 samples of gastric cancer tumor tissues, and 34 adjacent non-tumor tissue RNA sequence results were downloaded from the TCGA. Bioinformatics analyses showed that UCA1 was highly expressed in tumor tissues from gastric cancer patients. Shang et al. [12], Gu et al. [13] and Fang et al. [14] also reported that UCA1 was upregulated in gastric cancer tissues. However, little is known about the biological functions of UCA1 and its mechanism of action during targeted gene regulation.

In the present study, we characterized UCA1 tissue expression levels in 102 gastric cancer tumor tissues paired with adjacent non-cancerous tissues, and correlated these results with clinical features. In addition, based on the UCA1 expression of gastric cancer cells, we constructed overexpression systems and used silencing lentivirus vectors to investigate the biological function of UCA1 *in vitro* and *in vivo*. The PI3K-Akt-mTOR signaling pathway, which is usually abnormal in many human cancers such as pancreatic cancer, colon cancer, breast cancer, and lung cancer [15–18], was identified as a direct target. Furthermore, our previous studies using microarray screening and bioinformatics analyses to construct the lncRNA-mRNA co-expression network [10], showed that some of the gastric cancer-related mRNAs were co-expressed with UCA1 and were involved in the regulation of the PI3K-Akt-mTOR signaling pathway. We also characterized the effects of UCA1 on the PI3K-Akt-mTOR signaling pathway in gastric cancer. Taken together, the results helped to identify the function of UCA1 and its role in gastric cancer tumorigenesis.

## RESULTS

### The expression of UCA1 was upregulated and correlated with TNM stage and lymph node metastases

**Figure 1:**
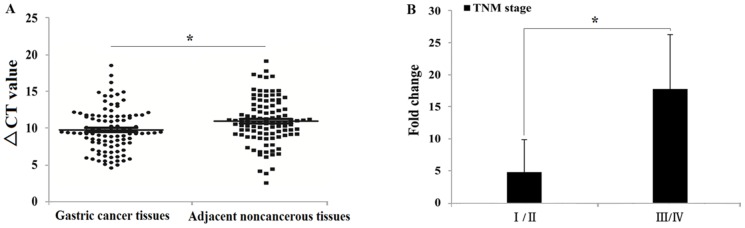
UCA1 expression level in 102 gastric cancer patients (A: Comparison between cancer tissues and adjacent noncancerous tissues, ΔCt = (Ct_RNAs_ – Ct _GAPDH_) ^*^P<0.05; B: Comparison of the UCA1 upregulation fold change between TNM stage I/II and III/IV, ^*^P<0.05).

The expression level of lncRNA UCA1 was determined in 102 paired gastric cancer tissues and adjacent non-cancerous tissues by qRT-PCR. In gastric cancer tissues, UCA1 expression was higher than the average level of adjacent non-cancerous tissues (Figure 1A). Analyses of the correlation between UCA1 expression and gastric cancer patients’ clinical pathological features showed that increased UCA1 expression was correlated with gastric cancer TNM stage and lymph node metastases, with no other correlation found between UCA1 expression and other gastric cancer clinical pathological features, including the patients’ sex, age, tumor size, and degree of differentiation (Table 1). In addition, we also compared the changes of UCA1 expression between the TNM (I/II) and TNM (III/IV) stages. The results showed that compared with adjacent non-tumor tissues, the expression of UCA1 showed a significant gradual increase from the TNM (I/II) to the TNM (III/IV) stages of gastric cancer (Figure 1B).

**Table 1:** The correlations between UCA1 and gastric cancer clinical features

Variable	Cases, n (%)	UCA1		Chi-squared test χ^2^-value	P-value
		Low-UCA1 group, no. of cases	High-UCA1 group, no. of cases		
Gender				0.000	1.000
Male	75 (74)	21	54		
Female	27 (26)	8	19		
Age, years				0.901	0.342
≤50	30 (29)	11	19		
>50	72 (71)	18	54		
Tumor size, cm				0.065	0.799
≤5	56 (55)	17	39		
>5	46 (45)	12	34		
Degree of differentiation				1.284	0.257
Well and moderately	35 (34)	7	28		
Poorly	67 (66)	22	45		
TNM stage				11.014	0.001^*^
I/II	60 (59)	25	35		
III/IV	42 (41)	4	38		
Lymph-node status				4.555	0.031^*^
No metastasis	48 (44)	19	29		
Metastasis	54 (56)	10	44		

lncRNA, long noncoding RNA; TNM, tumor node metastasis; ^*^ P<0.05.

### UCA1 was localized to the cytoplasm and was upregulated in gastric cancer cells

**Figure 2:**
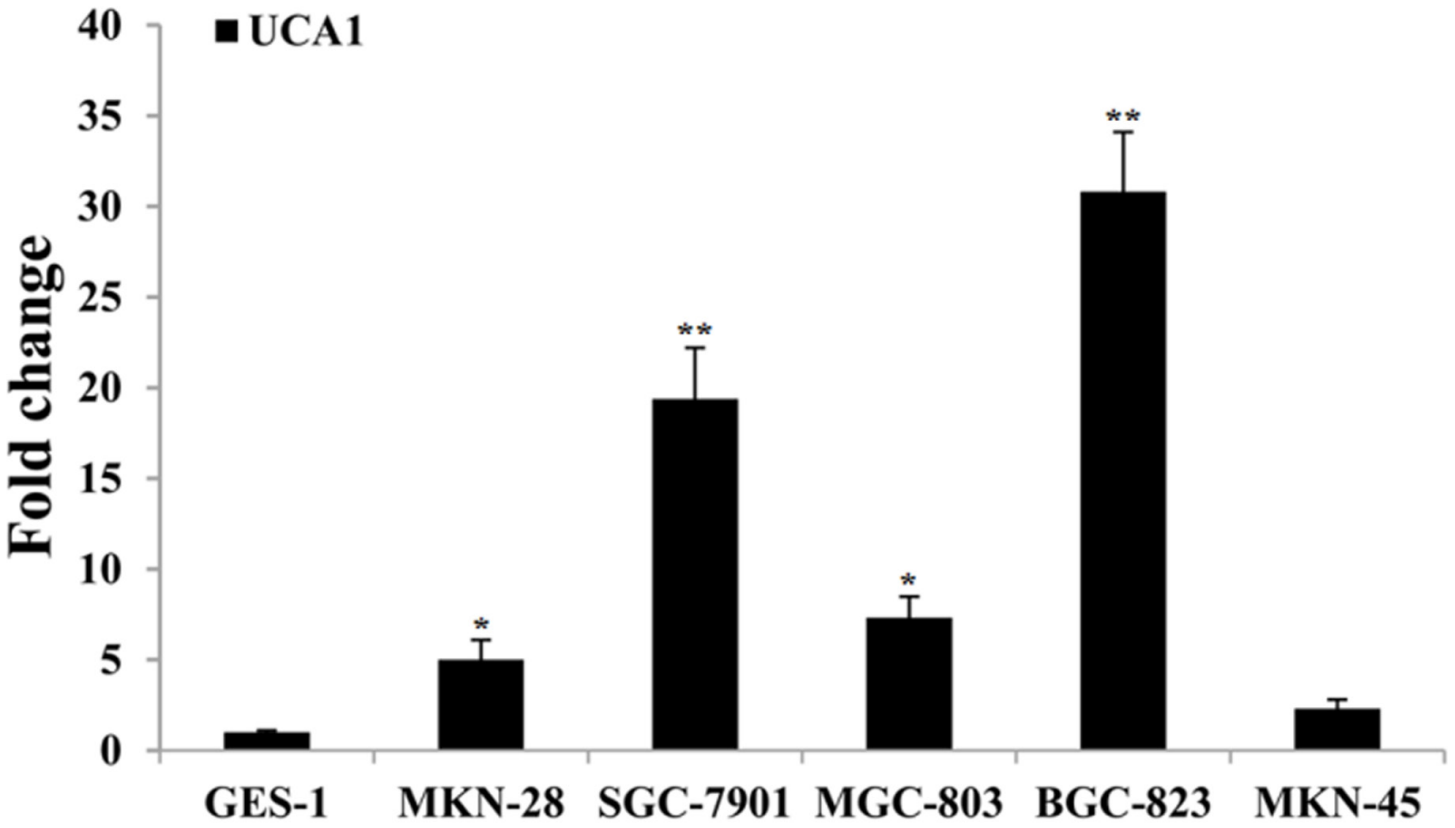
Results of UCA1 relative expression level between gastric cancer cells and normal gastric mucosa cell, respectively (^*^P<0.05; ^**^P<0.01).

**Figure 3:**
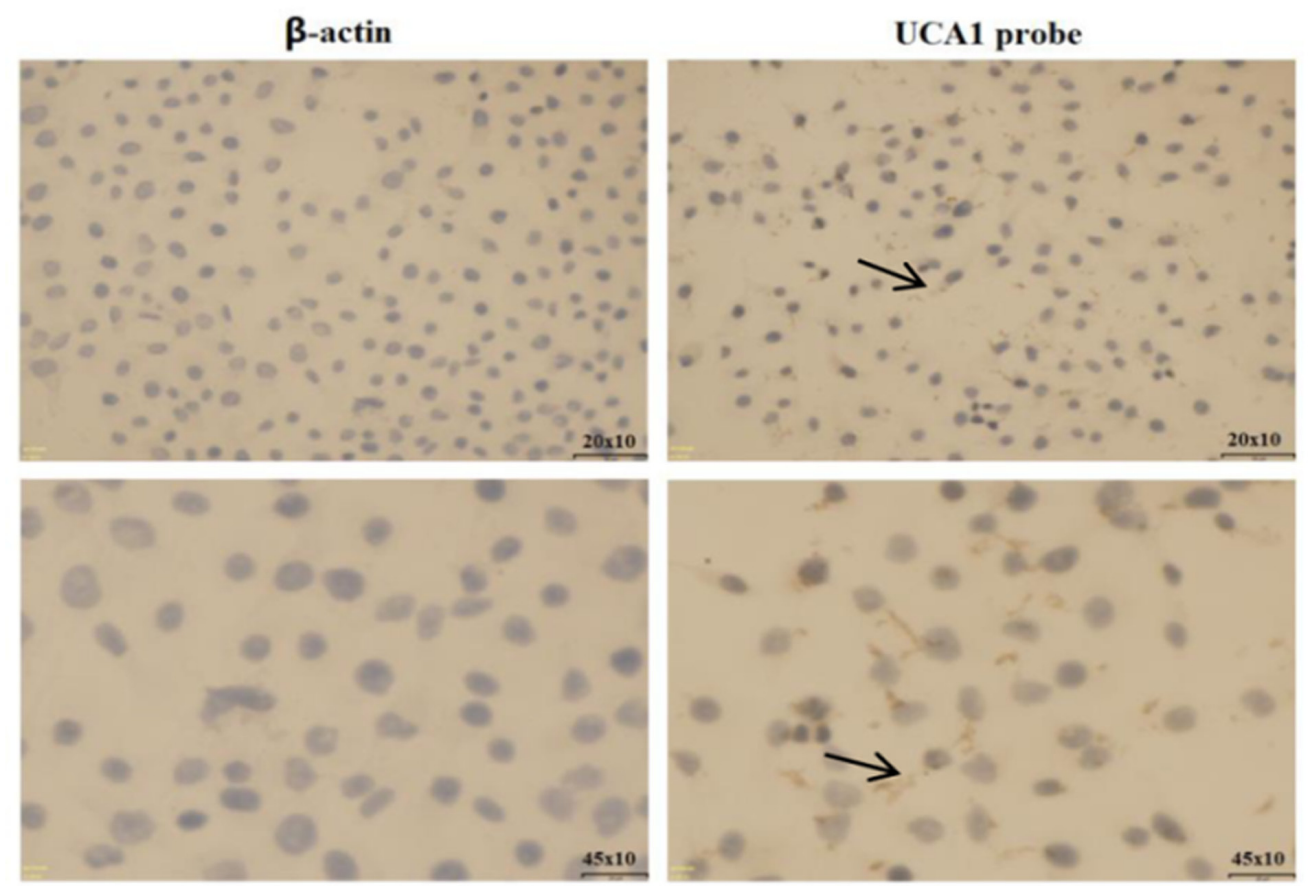
RNA situ hybridization assay of UCA1 in BGC-823 cells

The qRT-PCR was used to determine the expression of UCA1in five gastric cancer cell lines (MKN-28, SGC-7901, MGC-803, BGC-823, and MKN-45). The results showed that UCA1 expression was significantly upregulated in gastric cancer cell lines compared with the normal gastric epithelial cell line GES-1 (Figure 2). The most significantly upregulated gastric cancer cell line was BGC-823, with a 30.810-fold change of UCA1 when compared with that of the GES-1 cells (P < 0.01). Integrated analyses showed that UCA1 expression was frequently higher in gastric cancer tissues and gastric cancer cells. RNA *in situ* hybridization was then performed to determine the localization of UCA1 in BGC-823 cells. The results showed that UCA1 was found in the cytoplasm (Figure 3), and suggested that upregulation of UCA1 may play an important role in transcriptional and post-transcriptional regulation.

### Lentivirus-mediated infection altered the *in vitro* expression of UCA1

**Figure 4:**
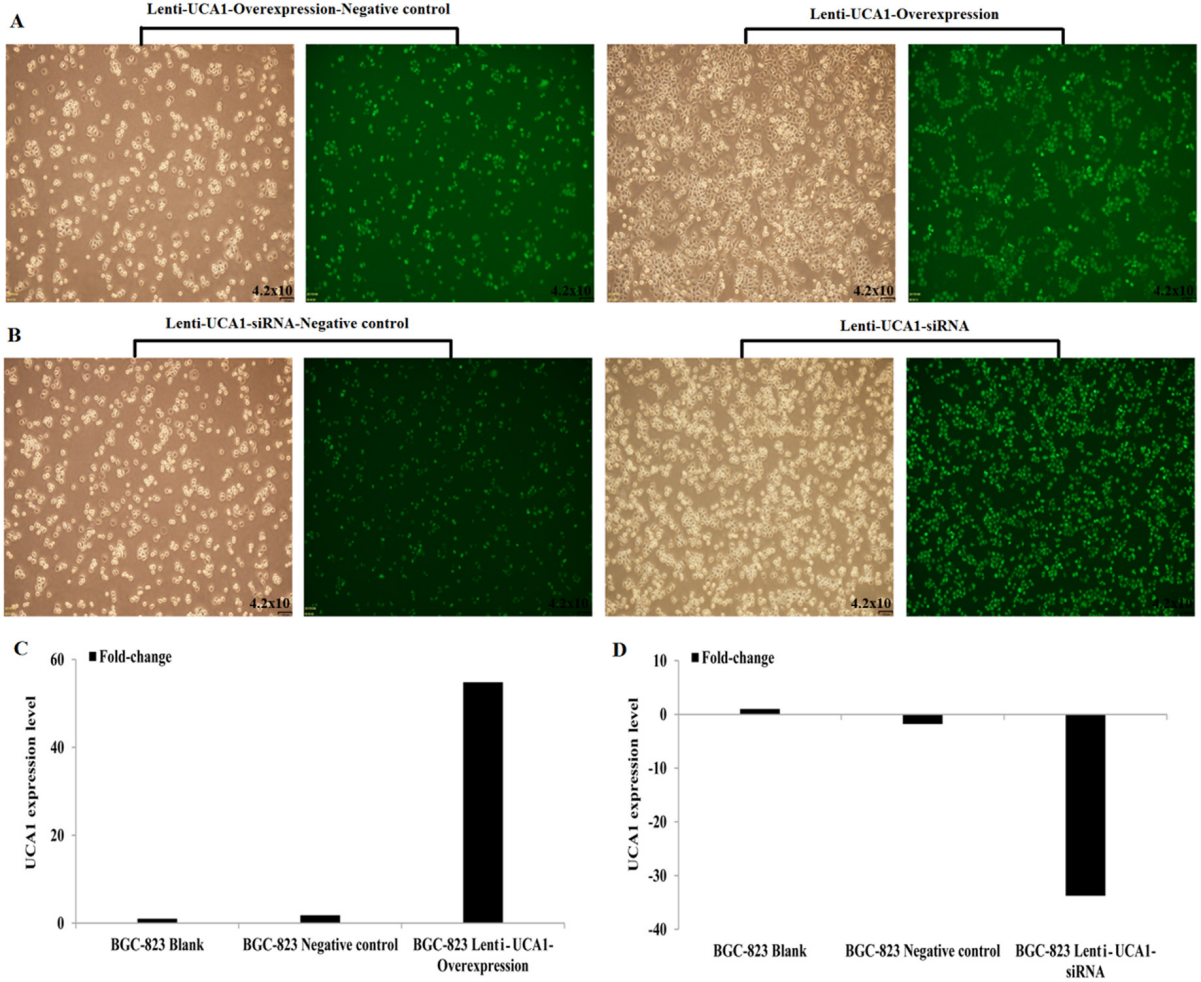
Lentivirus UCA1 overexpression and siRNA stabilization transfected BGC-823 cells efficiency **(A-B),** qRT-PCR detected **(C**: BGC-823 Lenti-UCA1-Overexpression; **D**: BGC-823 Lenti-UCA1- siRNA).

To assess the biological role of UCA1 in gastric cancer, we constructed lentivirus vectors that overexpressed UCA1 or an siRNA vector that downregulated UCAI expression, then used these vectors to infect the human gastric cancer cell line, BGC-823, according to the manufacturer’s instructions. Puromycin was then used to screen the stably-transfected cell lines. Figure 4A and 4B show that after puromycin screening, the stable infection efficiency approached 100%, as determined by the green fluorescent protein (GFP) expression. Quantitative real-time PCR (Figure 4C and 4D) showed that compared with the blanks and negative controls, in the lentivirus-UCA1-overexpression transfected BGC-823 cell line, the UCA1 expression was upregulated 54.82-fold; and in the lentivirus-UCA1-siRNA transfected cell line, BGC-823, the UCA1 expression level was downregulated 33.75-fold.

### The effect of UCA1 on gastric cancer cell proliferation and apoptosis *in vitro*

**Figure 5:**
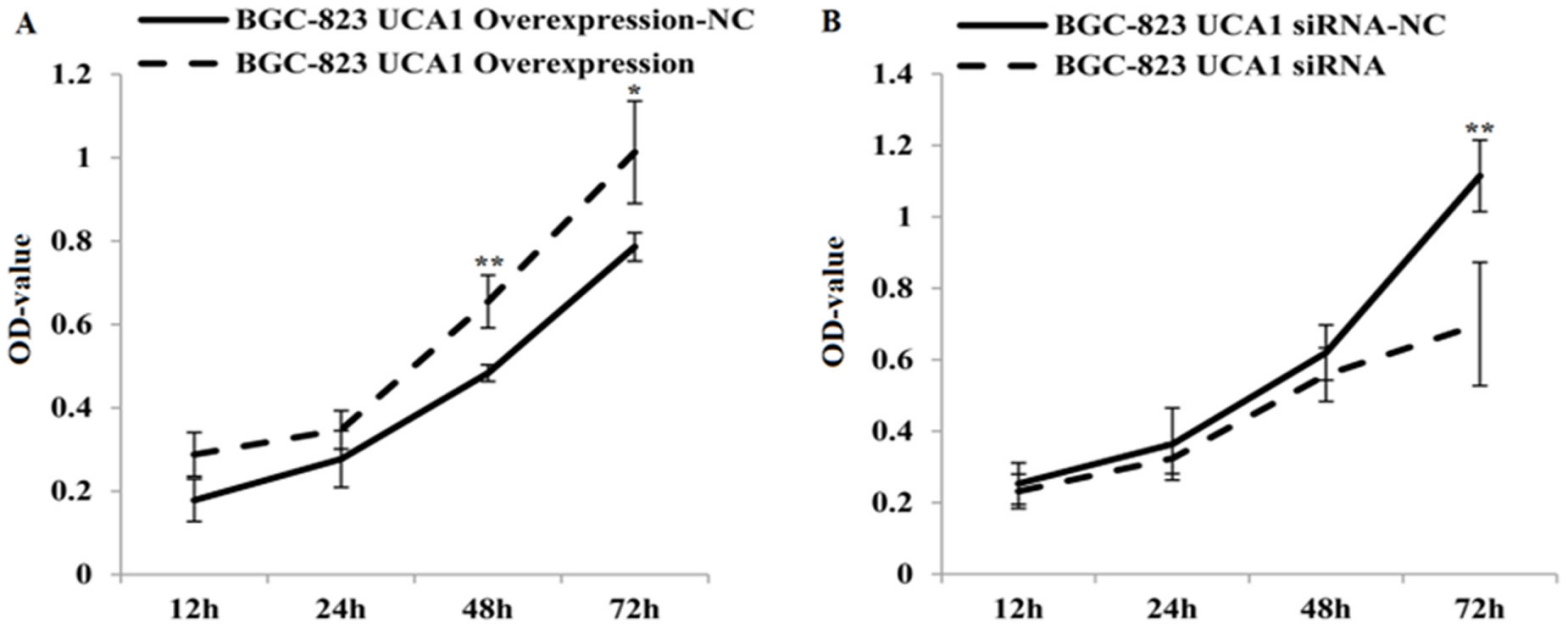
Lentivirus UCA1 overexpression and siRNA stable transfected BGC-823 cells, the proliferation with seeded and incubated for 12, 24, 48, 72h Cell proliferation was measured using a commercial MTT assay. Results show mean±SD of 3 independent experiments (^*^P<0.05, ^**^P<0.01; lentivirus UCA1 overexpression and siRNA stable transfected BGC-823 cells vs. negative control NC).

**Figure 6:**
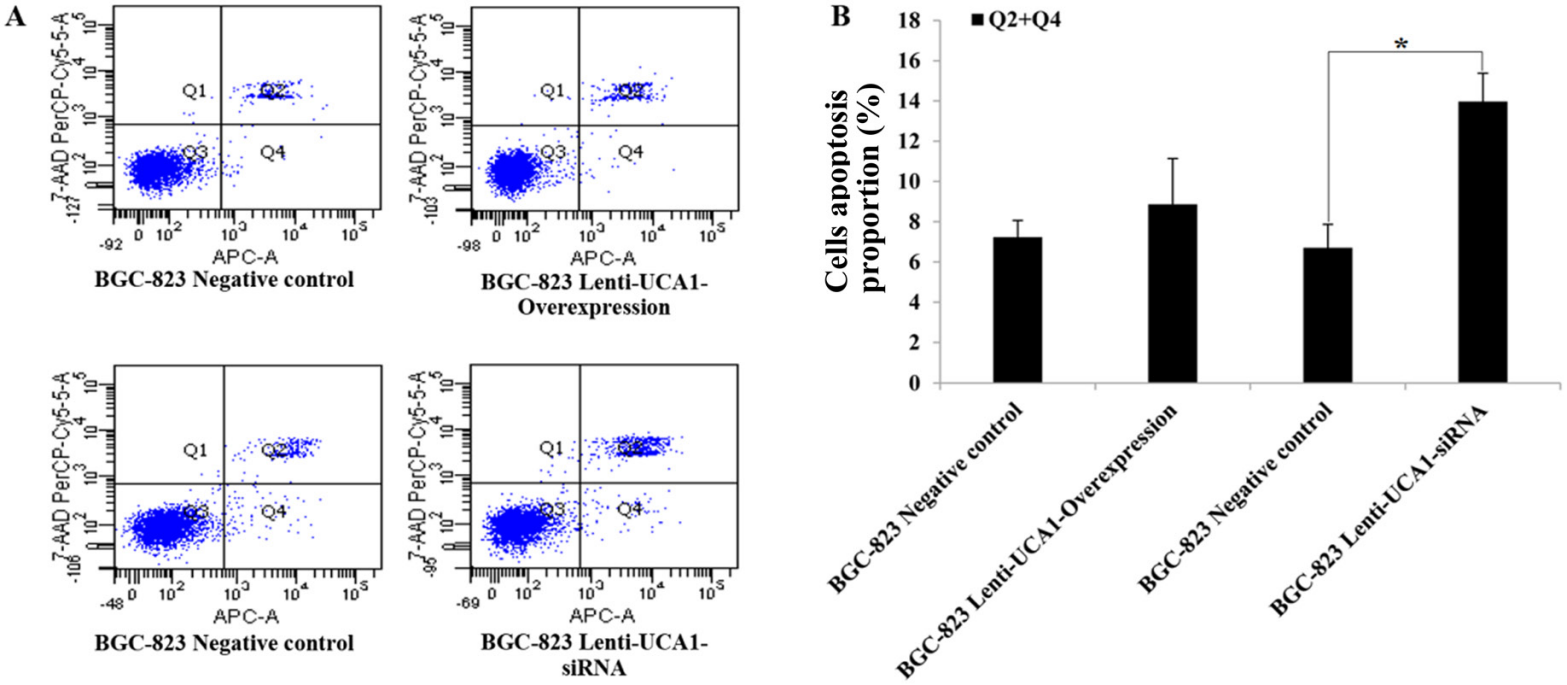
The effects of UCA1 on apoptosis in the lentivirus UCA1 overexpression and siRNA stable transfected BGC-823 cell via flow cytometry assay **(A)** Flow cytometry apoptosis assays results figures. **(B)** Transfected BGC-823 cell UCA1 overexpression and UCA1 siRNA vs. corresponding negative control. The values present mean±SD; (n=3) of the samples. (^*^P<0.05).

To investigate whether UCA1 could affect gastric cancer cell proliferation and apoptosis, we used an 3-(4, 5-dimethylthiazol-2-yl)-2, 5-diphenyltetrazolium bromide (MTT) cell proliferation assay and a flow cytometry assay to detect lentivirus-UCA1 overexpression and siRNA vectors that were used to transfect BGC-823 cells. The MTT assay results showed that BGC-823 cell growth was significantly decreased in the lentivirus-UCA1-siRNA cells after 48 hours when compared with the negative control cells (P < 0.01; Figure 5B). In the lentivirus-UCA1 overexpression cells, cell growth increased after 48 hours when compared with the negative control cells (P < 0.05; Figure 5A). We also used flow cytometry to determine apoptosis of the stably transfected BGC-823 cells (Figure 6), and found that BGC-823 cell apoptosis was significantly increased after lentivirus-UCA1-siRNA transfection when compared with the negative control cells (Figure 6B).

### UCA1-siRNA decreased the migration and invasion of gastric cells

**Figure 7:**
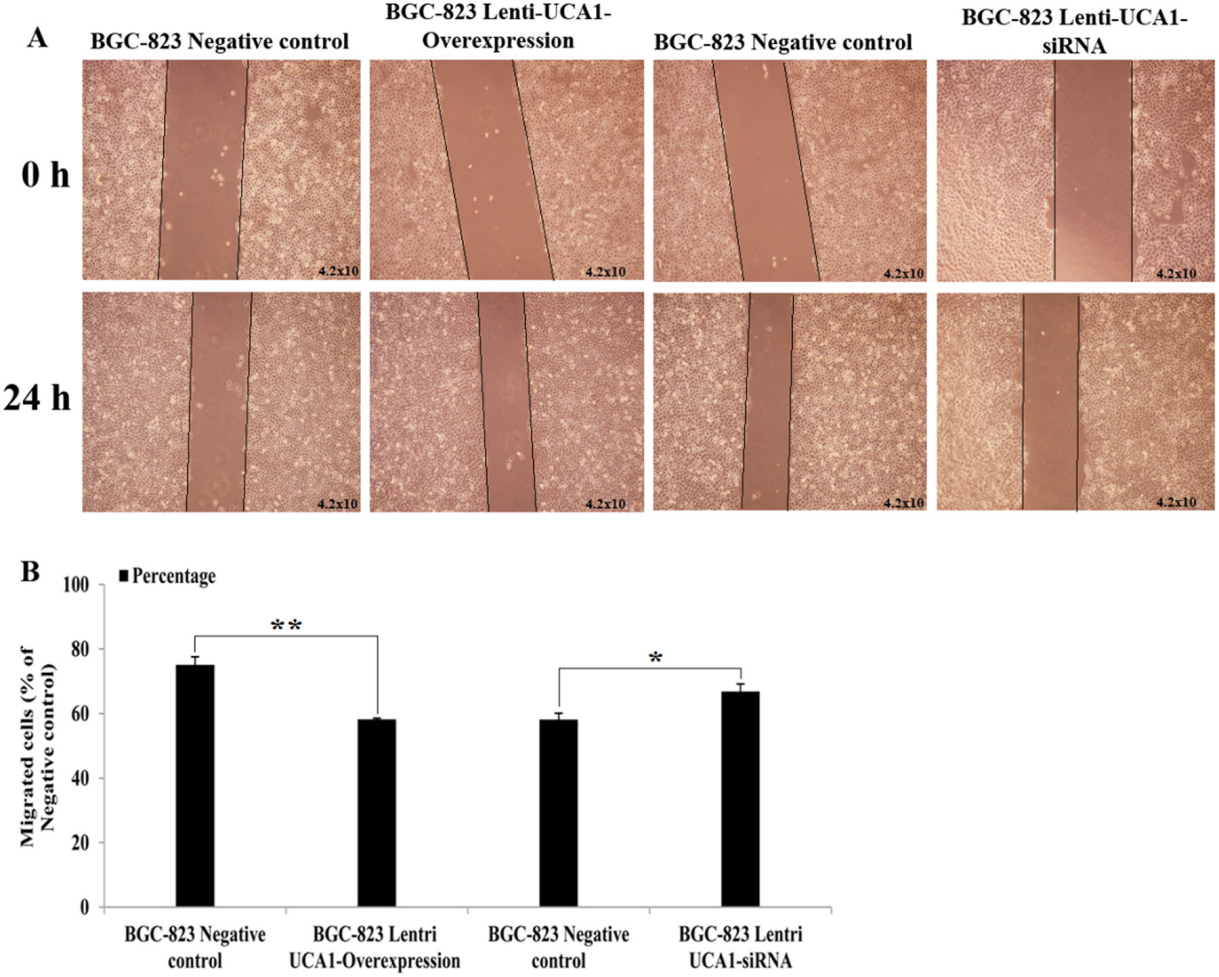
**(A)** Representative images of BGC-823 cell lentivirus UCA1 overexpression and siRNA stable transfected at 0 and 24 h after wound scratch in wound-healing assa. **(B)** Image pro plus was used to analyze the results of 0 and 24 h after wound scratch images (^*^P<0.05, ^**^P<0.01).

**Figure 8:**
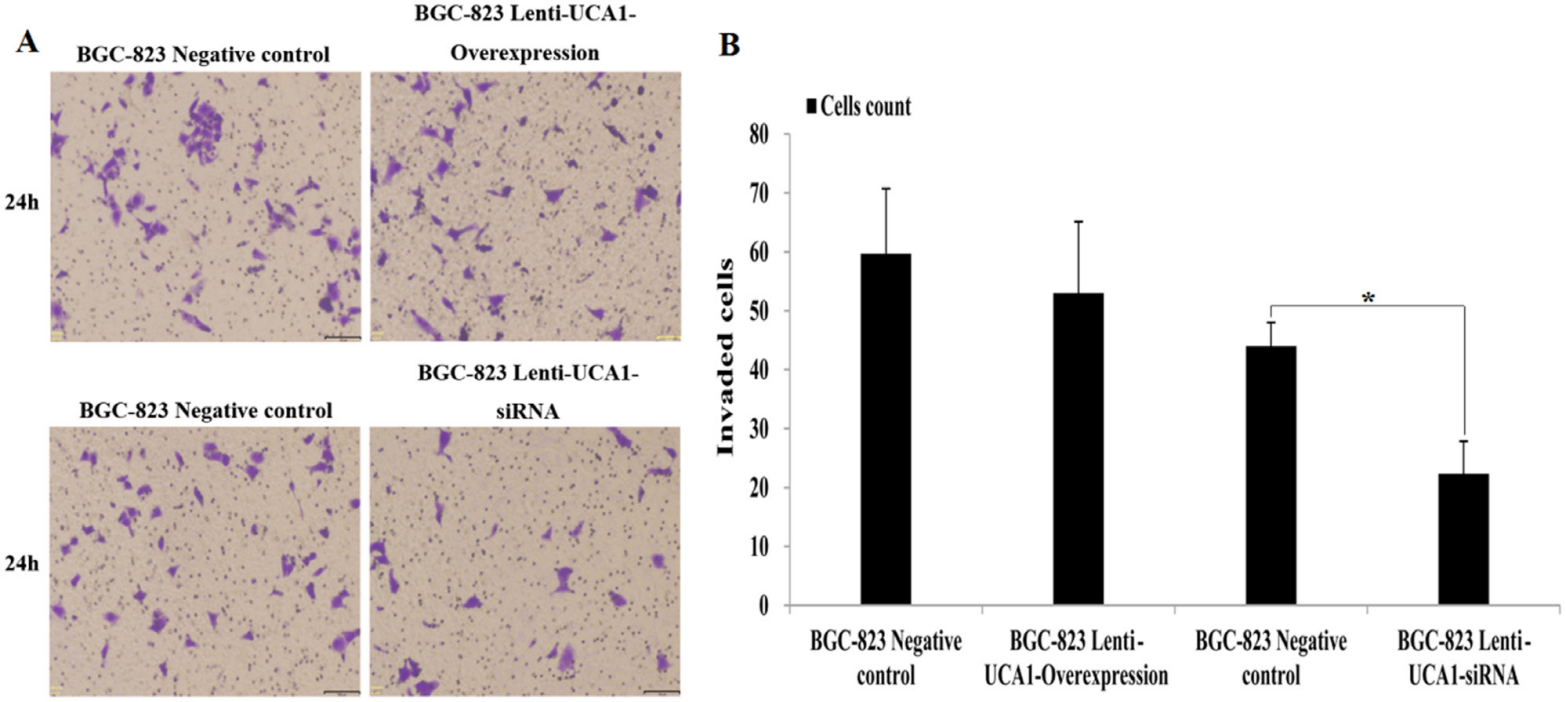
**(A)** The representative images of crystal violet stained for lentivirus UCA1 overexpression and siRNA stable transfected of BGC-823 cell in trans-well invasion assays. **(B)** The values present mean±SD; (n=6) of the microscopic fields. ^*^P<0.05.

Using gastric cancer patients’ tumor tissues and paired adjacent non-cancerous tissues, the expression of UCA1 was shown to be highly correlated with the TNM stage and metastatic properties. We also determined whether UCA1 played an important role in migration and invasion of gastric cancer. A wound healing assay and the Transwell^®^ invasion assay were used to detect gastric cancer cell migration and invasion capability using UCA1 gene overexpression and interference techniques. As shown in (Figure 7A and 7B), compared with negative control the migration increased significantly in BGC-823 cells when stably infected with lenti-UCA1-overexpression, and the percentage of wound scratch gap closures was significantly faster than that of the negative control group (P < 0.01). In addition, the migration decreased significantly in BGC-823 cells when stably infected with lenti-UCA1-siRNA, and the percentage of wound scratch gap closures was significantly slower than that of the negative control group (P < 0.05). The Transwell^®^ assay also showed similar results after stable infection with the lenti-UCA1-siRNA. UCA1 lenti-UCA1-siRNA stable infection lowered the number of BGC-823 invading cells, as shown by the decrease in the percentages of invasion when compared with the negative control group (Figure 8). Taken together, the results showed that UCA1 silencing inhibited cell migration and invasion in gastric cancer cells.

### UCA1 regulated PI3K-Akt-mTOR signaling proteins and their downstream mediators

**Figure 9:**
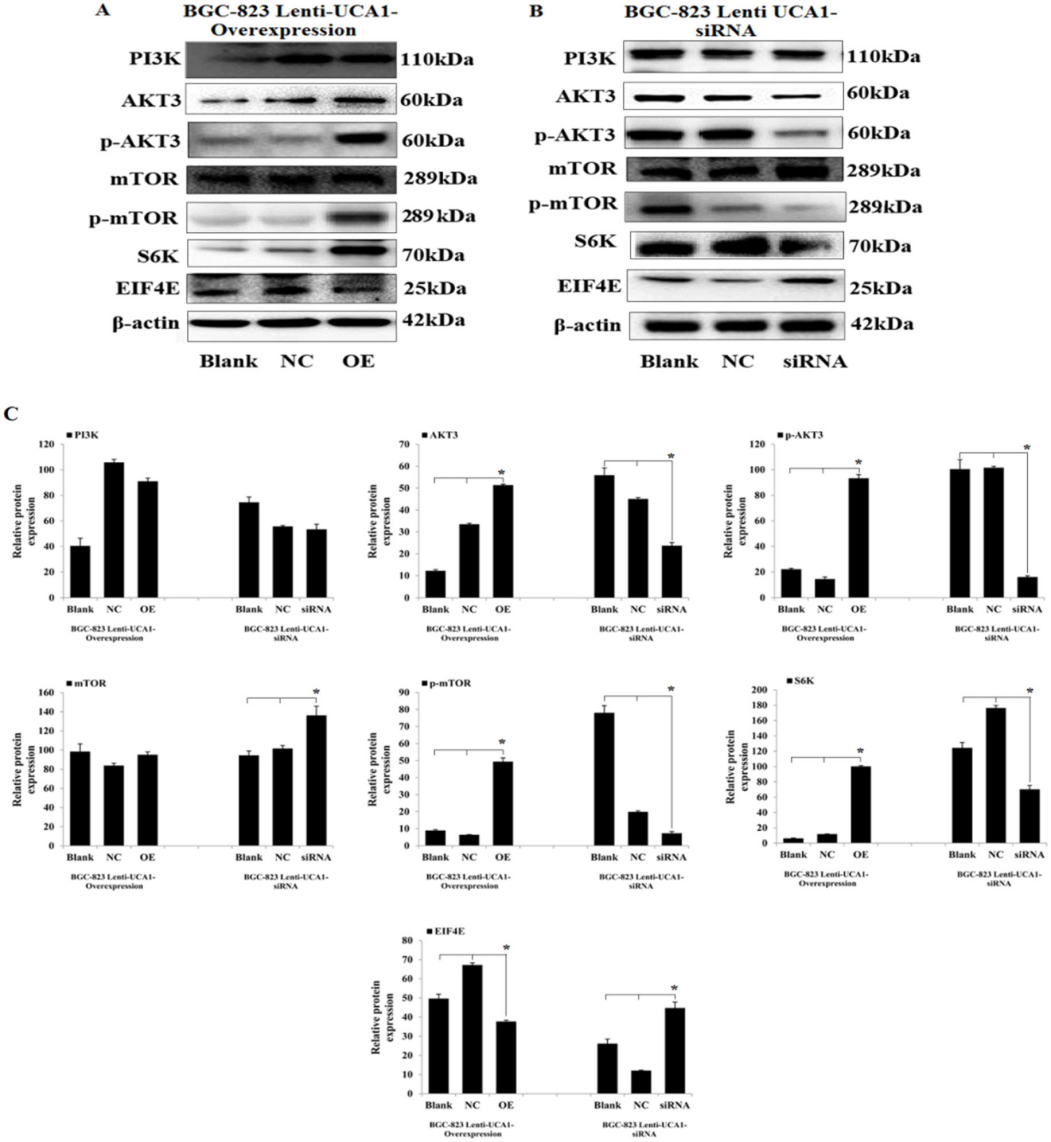
UCA1 effects gastric cancer malignant progression through PI3K-Akt-mTOR signaling pathway Representative western blotting results for PI3K, AKT3, p-AKT3, mTOR, p-mTOR, S6K and EIF4E protein expression from Lenti-UCA1 overexpression **(A)** and siRNA **(B)** stable transfected BGC-823 cells. **(C)** Western blotting results analyzed of relative optical density in protein expression by Image pro plus software. The relative protein expression levels were obtained from three independent experiments, β-actin was used as a control, mean ± SD, ^*^P < 0.05.

**Figure 10:**
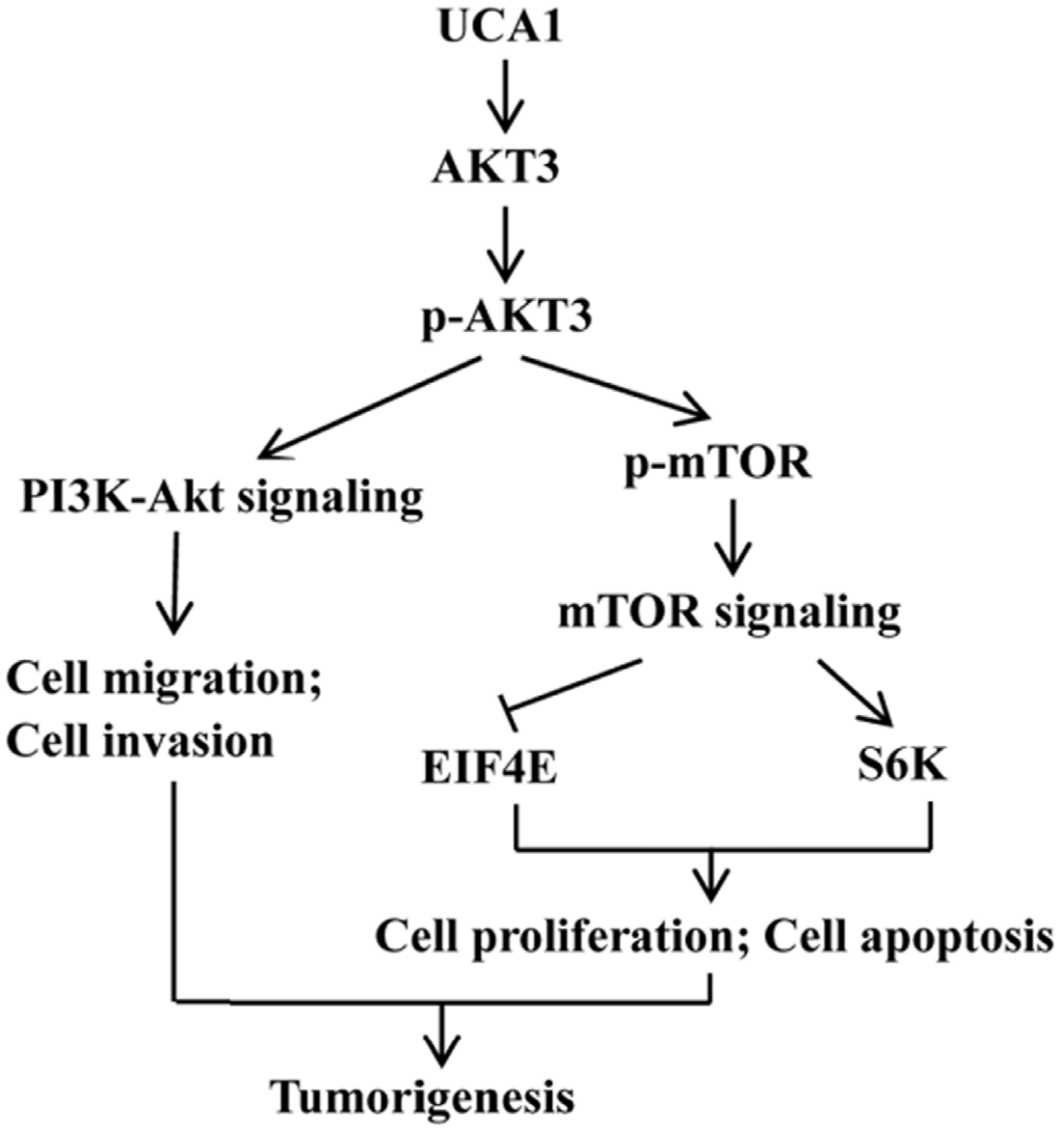
Diagram depicting the regulation mechanism of UCA1 in the tumorigenesis of gastric cancer

PI3K-Akt-mTOR is an important signaling pathway that affects cell energy metabolism, proliferation, apoptosis, the cell cycle, cell size, and cell invasion and survival times, and is closely associated with many types of cancers [19]. Our previous studies [10] found that some of the gastric cancer-related mRNAs were co-expressed with UCA1 and were involved in the PI3K-Akt-mTOR signaling pathway, so we investigated whether the induction of proliferation, apoptosis, and invasion in gastric cancer cells by UCA1 involved regulation of the key PI3K-Akt-mTOR signaling proteins and their downstream mediators. Cell lysates were prepared from BGC-823 cells that were stably transfected by lentivirus causing UCA1 overexpression, by siRNA, and by a negative control, followed by western blot analyses. The results showed that UCA1 overexpression increased the expression of AKT3, p-AKT3, p-mTOR, and S6K, and inhibited the expression of EIF4E in BGC-823 cells, when compared with the blank and negative controls, respectively (P < 0.05; Figure 9A and 9C). In addition, stable infection with lenti-UCA1-siRNA inhibited the expression of AKT3, p-AKT3, p-mTOR, and S6K, and increased the expression of EIF4E in BGC-823 cells, when compared with the blank and negative controls (P < 0.05: Figure 9B and 9C). Together, the resulted showed that UCA1 affected malignant progression of gastric cancer cells partly through p-AKT3 and p-mTOR in the PI3K-Akt-mTOR signaling pathway (Figure 10).

### UCA1-siRNA inhibited gastric cancer cells tumorigenesis *in vivo*

**Figure 11:**
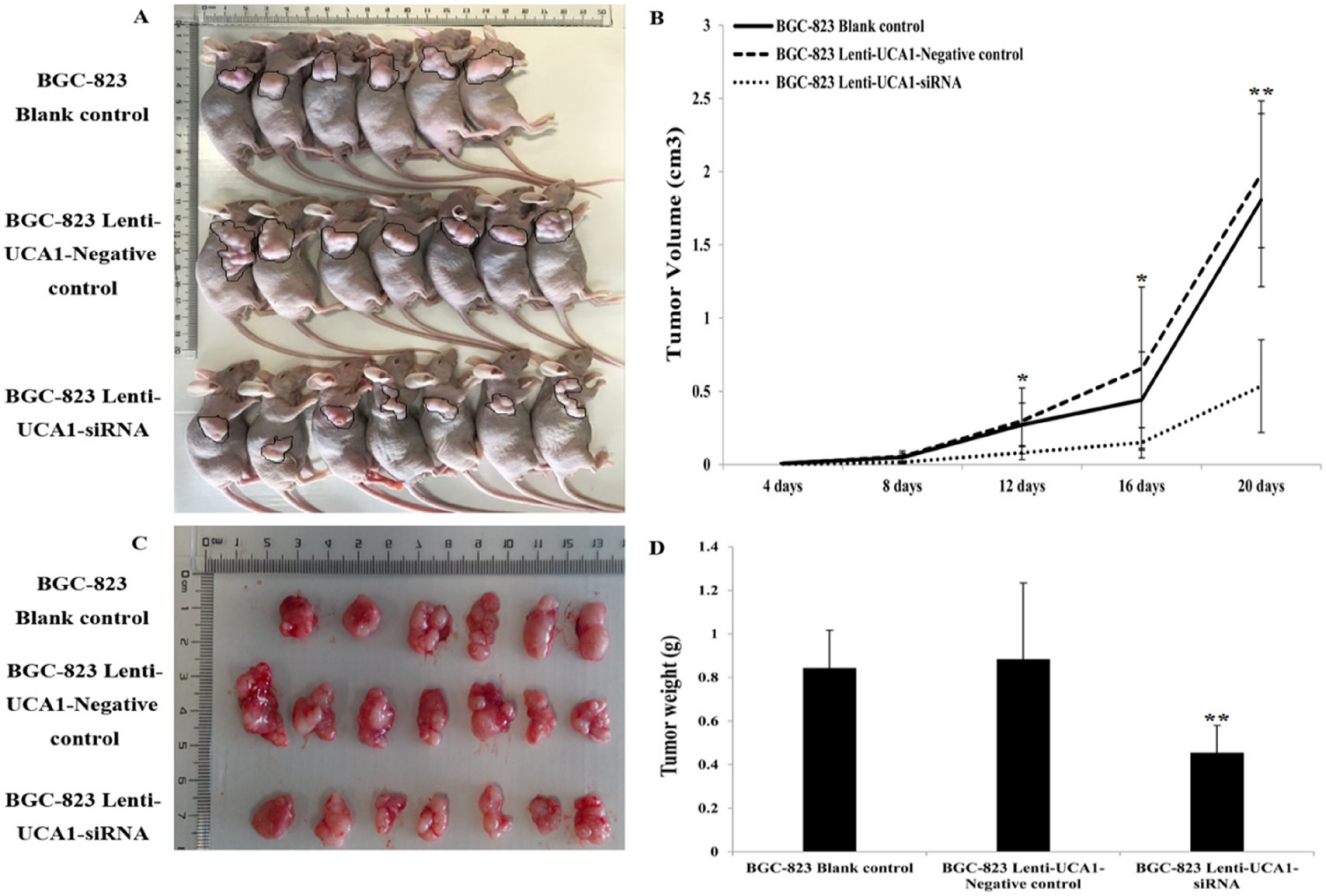
Tumor mass after injection into subcutaneous of nude mice **(A)** Effects of UCA1 on tumor growth *in vivo*. **(B)** The volume of the subcutaneous tumors was measured every 4 days for 20 days. The tumor growth in BGC-823 blank control and lenti-UCA1-negative control were significantly impaired in lenti-UCA1-siRNA group 16 days after subcutaneous injection (P<0.01). **(C-D)** Dissected tumor tissues from nude mice. BGC-823 lenti-UCA1-siRNA group the tumors are smaller than those from the blank control and negative control groups.

**Figure 12:**
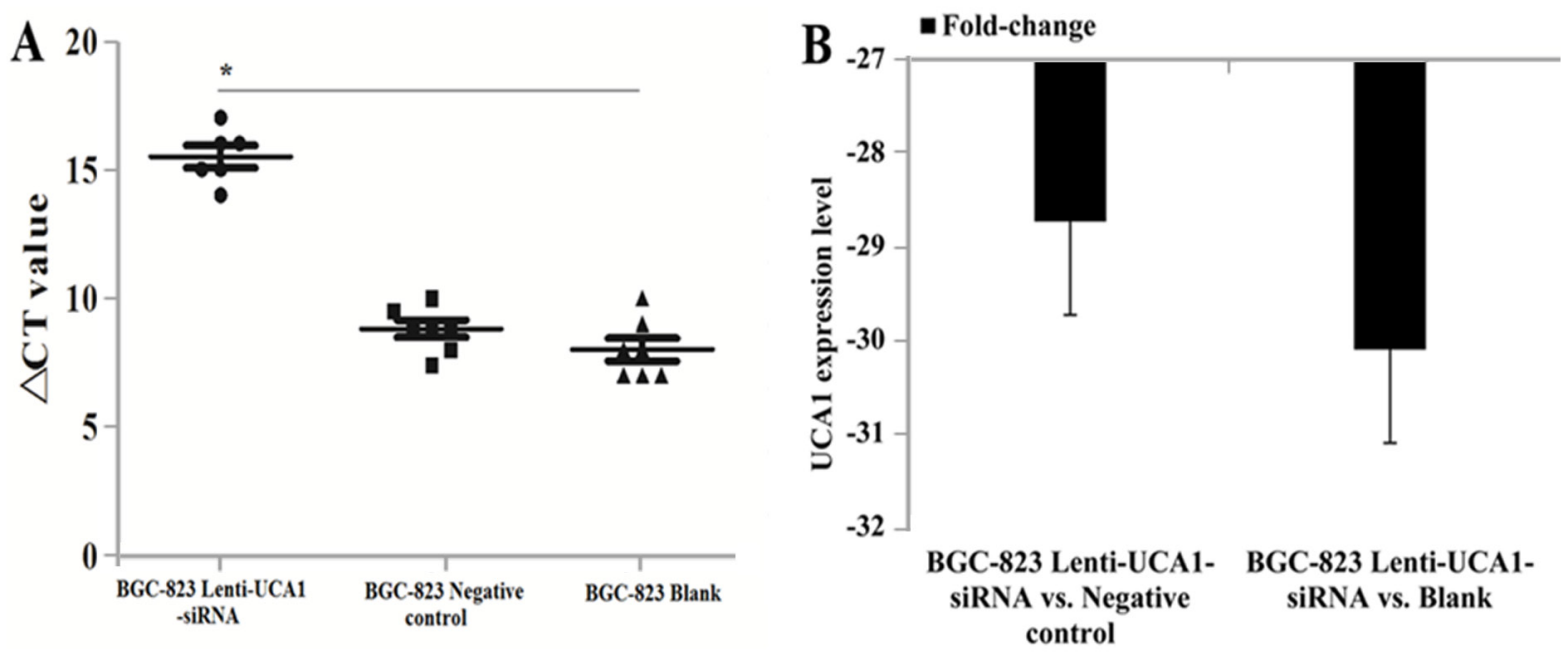
Tumor tissue UCA1 expression levels detection using qRT-PCR *in vivo* of nude mice. **(A)** ∆CT value comparision between BGC-823 lenti-UCA1-siRNA, negative control and blank groups. **(B)** The UCA1 expression levels fold change between BGC-823 lenti-UCA1-siRNA vs. negative control and BGC-823 lenti-UCA1-siRNA vs. blank.

**Figure 13:**
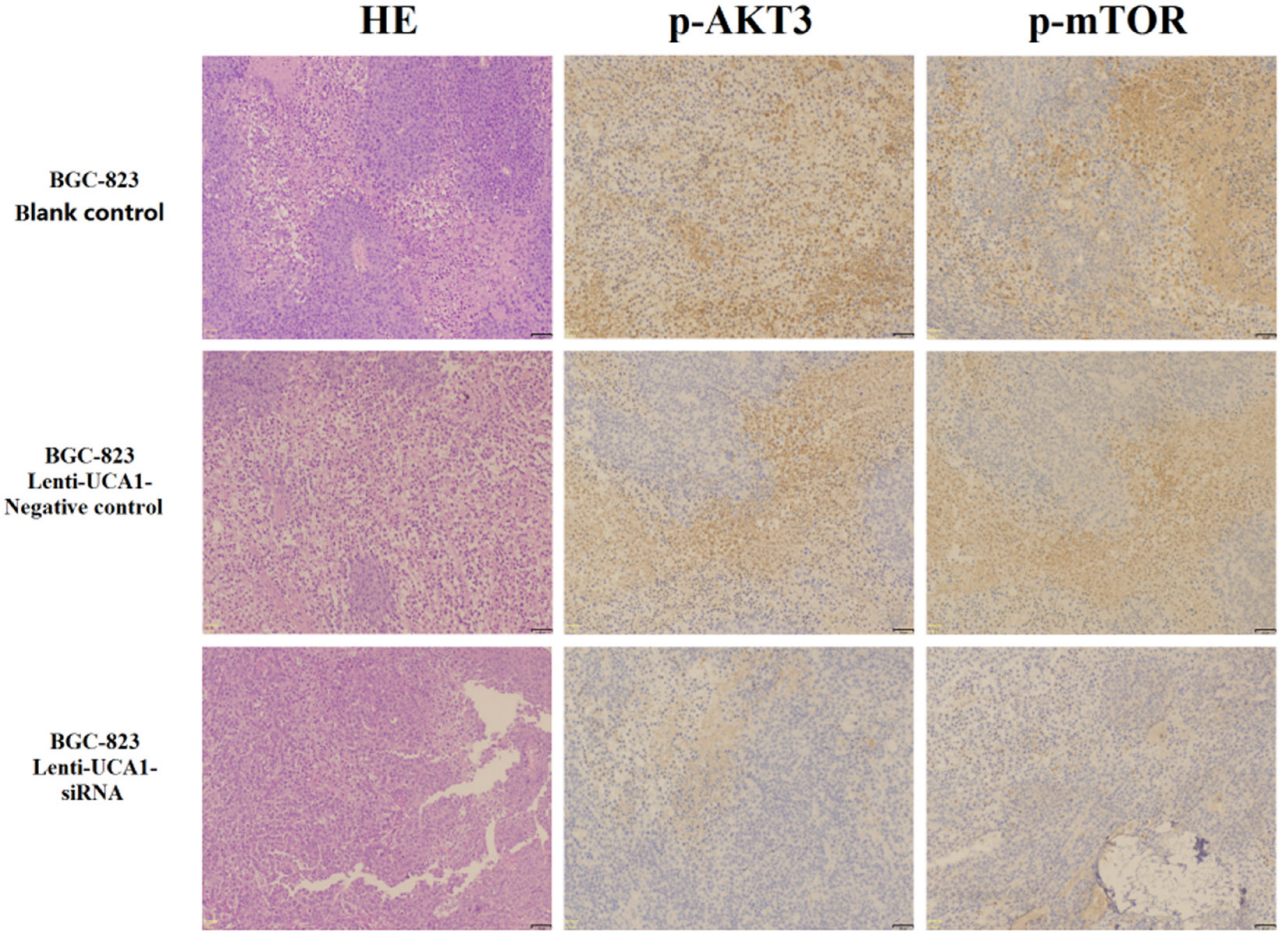
Immunohistochemical technique analysis the tumor tissue proteins expression levels of p-AKT3 and p-mTOR *in vivo* of nude mice

To explore whether the level of UCA1 expression affected gastric cancer tumorigenesis, lenti-UCA1-siRNA and empty vector stably-transfected BGC-823 cells were inoculated into three groups of male nude mice. Twenty days after injection, the tumors formed in the UCA1 silencing group were substantially smaller than those in the blank group and the negative control group (Figure 11A and 11B). Moreover, the average tumor weight at the end of the experiment was significantly lower in the UCA1 silenced group (0.46 ± 0.12 g) compared to the blank group (0.84 ± 0.17g) and the empty vector group (0.88 ± 0.35g), (P < 0.05; Figure 11C and 11D). Quantitative RT-PCR analyses of UCA1 expression were performed in isolated tumor tissues, showing that UCA1 expression in tissues formed from UCA1 silenced cells were lower than those of tumors formed by the blank and negative cells (Figure 12A and 12B). Immunohistochemistry was used to analyze the p-AKT3 and p-mTOR protein levels in isolated tumor tissues. The results suggested that in tumors produced by the lenti-UCA1-siRNA stably-transfected BGC-823 cells, the protein levels of p-AKT3 and p-mTOR were significantly inhibited when compared with the tumors from the BGC-823 blank and negative control cells (Figure 13). Taken together, the results showed that silencing of UCA1 inhibited gastric cancer tumorigenesis progression *in vivo*.

## DISCUSSION

Gastric cancer currently remains a major public health problem throughout the world. Despite improvements in gastrointestinal endoscopy and surgery, the prognoses of advanced gastric cancer patients remain poor, with a 5-year survival percentage of < 30% [20–22]. Studies of the molecular mechanisms involved in different stages of gastric cancer are urgently needed for the development of better methods of diagnoses and improvements in therapeutic treatments for this disorder. LncRNAs are components of the tumor process that are involved in tumor suppression or oncogenesis through the regulation of gene expressions at the transcriptional and post-transcriptional levels [23]. UCA1 is located on chromosome 19p13.12 and was reported to be highly expressed in bladder cancer cells and to be involved in promoting migration and invasion during tumorigenesis [24, 25]. In addition, Fotouhi et al [26], Guo et al [27] and Xu et al [28] reported that UCA1 was aberrantly expressed in prostate cancer, lung cancer, and breast cancer. Our previous studies have also reported that UCA1 was significantly differentially expressed in gastric cancer tumor tissues and their paired adjacent non-tumor tissues [10]. It is therefore assumed that lncRNA UCA1 plays an important role in tumorigenesis, although the mechanism of action is still unclear.

In the present study, we determined the UCA1 expression in 102 gastric cancer tumor tissues and their paired adjacent non-cancerous tissues, and correlated these results with the clinical features of the tumors. The qRT-PCR results showed that UCA1 expression was increased in gastric cancer tumor tissues. Zheng et al. [29] and Gao et al [30] also reported that UCA1 was overexpressed in gastric cancer tissues and cell lines compared with that in normal control tissues. Together, the results suggested that UCA1 may function as an oncogene in gastric cancer progression. Subsequently, we compared UCA1 expression and gastric cancer patients’ clinical pathological features, and showed that UCA1 expression was correlated with the gastric cancer TNM stage and lymph node metastases, but was not correlated with other gastric cancer clinical pathological features. Zheng et al. [29] also reported that during gastric cancer, high UCA1 expression correlated with tumor size, invasion depth, worsening differentiation, TNM stage, and the overall survival. UCA1 is therefore a potential oncogene that may play an important regulatory role in the clinical progression of gastric cancer.

To identify the biological functions of UCA1 in gastric cancer, we selected the high expression BGC-823 cell line. A RNA *in situ* hybridization assay was then performed to determine the localization of UCA1 in BGC-823 cells. The results showed that UCA1 was localized to the cytoplasm, suggesting that upregulation of UCA1 may play an important role in transcriptional and post-transcriptional regulation. We then determined whether UCA1 affected the functions of gastric BGC-823 cancer cells, using lentivirus UCA1 overexpression and siRNA stably-transfected cells. The MTT cell proliferation assay and flow cytometry both suggested that lentivirus-UCA1-siRNA stably-transfected BGC-823 cells showed significantly decreased proliferation and increased cell apoptosis of gastric cancer BGC-823 cells. Shang et al. [31] also reported that cell proliferation of gastric cancer cells was significantly inhibited by silencing of UCA1. Together, these results showed the *in vitro* effects of UCA1 on gastric cancer cell proliferation and apoptosis; however, the relationship between the expression of UCA1 and gastric cancer progression still remains unclear.

The results of the present study showed that the expression of UCA1 was significantly different during a gastric cancer TNM stage and lymph node metastases level, suggesting that high expression of UCA1 may play a role in activation of gastric cancer invasion and metastasis. Wang et al. [32] reported that UCA1 silencing inhibited the growth and metastasis of hepatocellular carcinoma cell lines *in vitro* and *in vivo*. Xue et al. [33], Li et al. [34] and Bian et al. [35] reported similar findings during the invasion and metastasis in bladder cancer, osteosarcoma, and colorectal cancer. Zuo et al.[36] reported that UCA1 Upregulation can promotes gastric cancer migration and invasion. In addition, metastasis studies of UCA1 during gastric cancer have rarely been reported [37, 38]. Therefore we determined whether UCA1 played an important role in migration and invasion of gastric cancer cells *in vitro*. Based upon the wound healing assay and Transwell^®^ invasion assay results, we found that migration increased in BGC-823 cells when stably infected with lenti-UCA1-overexpression, and decreased when stably infected with lenti-UCA1-siRNA, compared with their corresponding negative controls. In a similar manner, the number of BGC-823 invading cells decreased after stable infection with lenti-UCA1-siRNA. Taken together, the results showed that UCA1 silencing inhibited BGC-823 cell migration and invasion, suggesting that UCA1 can be used as a gene-specific target for gastric cancer treatment.

To gain insight into the molecular mechanism by which UCA1 contributes to cell proliferation, apoptosis, migration, and invasion of gastric cancer, we determined the expression of potentially-related proteins that were responsible for the above parameters. According to our previous study [10], some of the gastric cancer-related mRNAs were co-expressed with UCA1 and were involved in the PI3K-Akt-mTOR signaling pathway. PI3K-Akt-mTOR signaling pathway including PI3K, AKT, PTEN and mTOR etc. signaling key molecules. S6K and EIF4E are the downstream regulated target molecules of mTOR signaling pathway. S6K can be activated by phosphorylation mTOR then promote the occurrence of the ribosome. Meanwhile, EIF4E was inhibited due to the phosphorylation of mTOR. S6K and EIF4E combined and effect on the protein synthesis, regulating cell growth, apoptosis, and other important process of cell biology. The present study confirmed that UCA1 overexpression increased the expression of AKT3, p-AKT3, and p-mTOR, S6K, and inhibited the expression of EIF4E in BGC-823 cells, when compared with blank and negative control cells. Furthermore, stable infection with lenti-UCA1-siRNA inhibited the expression of AKT3, p-AKT3, p-mTOR, and S6K, and increased the expression of EIF4E in BGC-823 cells, when compared with blank and negative control cells. We also determined tumor tissue protein expression of p-AKT3 and p-mTOR using an immunohistochemical assay of tissues from nude mice. The results also suggested that lenti-UCA1-siRNA stably-transfected BGC-823 cells significantly inhibited p-AKT3 and p-mTOR protein expression in isolated tumor tissues of these animals. Thus, based on the expression of key proteins in the PI3K-Akt-mTOR signaling pathway, UCA1 overexpression activated the PI3K-Akt-mTOR signaling pathway and UCA1 silencing inhibited the PI3K-Akt-mTOR signaling pathway. The PI3K-Akt-mTOR signaling pathway plays an important role in the carcinogenesis of common cancers [39, 40]. In the present study, we showed that the dysregulated expression of UCA1 affected the expression of its key protein targets, such as p-AKT3, p-mTOR, and S6K, which are involved in the regulation of many biological processes during carcinogenesis, including cell proliferation, cell apoptosis, cell migration, and invasion. There have been several reports that UCA1 affected AKT expression and the activity of the PI3K-Akt-mTOR signaling pathway in the progression of bladder carcinoma cells [41], prostate cancer [42], breast cancer [43] and non-small cell lung cancer [44], but similar studies of gastric cancer have not been reported.

Finally, we also characterized the tumorigenesis and expression of UCA1, p-AKT3, and p-mTOR using lenti-UCA1-siRNA *in vivo*. The results showed that tumors formed in UCA1 silenced male nude mice were smaller than those in the blank mice and negative control mice. The results of our previous functional assays were also consistent with the role of UCA1 silencing in the inhibition of tumorigenesis. Similar studies of UCA1 have also been reported in other cancers involving the knockdown of UCA1, and resulting in the inhibition of bladder cancer proliferation *in vivo* [45]. Together, our results showed that silencing of UCA1 inhibited gastric cancer tumor growth *in vivo*.

In conclusion, the present study helped to define the role of UCA1 in gastric cancer tumorigenesis. We showed that UCA1 expression was significantly upregulated in gastric cancer tissues and cell lines, and the upregulated UCA1 correlated with the gastric cancer TNM stage and lymph node metastases. Furthermore, a functional assay indicated that UCA1 silencing significantly inhibited gastric cancer BGC-823 cell proliferation and increased apoptosis. We also showed that UCA1 might play an important role in the migration and invasion of gastric cancer cells *in vitro* and *in vivo*. The study of the molecular mechanism of UCA1 suggested that UCA1 regulates PI3K-Akt-mTOR signaling proteins and their downstream mediators, and alters gastric cancer progression *in vitro* and *in vivo*. To the best of our knowledge, this study is the first to systematically characterize the relationship between UCA1 and gastric cancer. As a proof of the functional importance of lncRNA, we showed that gastric cancer development and progression were potentially regulated by UCA1 expression. Based upon these results, we suggest that UCA1 can be a novel target for therapeutic intervention of gastric cancer.

## MATERIALS AND METHODS

### Tissue collection

A total of 102 gastric cancer samples were collected from patients who underwent surgery at Wuwei Tumor Hospital of Gansu (Wuwei, China), between 2014 and 2016. These patients were diagnosed with gastric cancer (stage II, III, and IV) based on the seventh edition of the American Joint Committee on Cancer (AJCC) Cancer Staging Manual. Clinical information recorded for each specimen included age, tumor grade, cancer stage, tissue dimensions, and lymphatic metastases status. The data were available for all samples (Table 1). No local or systemic treatment was conducted in these patients before surgery. None of the patients received preoperative chemoradiation. Adjacent non-cancerous tissues were located ≥ 5 cm from the tumor edge. Tissue samples were immersed in RNAlater^®^ (Ambion, Austin, TX, USA) and stored at −80°C until use. The study was approved by the Research Ethics Committee of Wuwei Tumor Hospital of Gansu (Wuwei, China). Informed consents were obtained from all patients.

### Gastric cancer cell lines and culture conditions

Five gastric cancer cell lines (MKN-28, SGC-7901, BGC-823, MGC-803, and MKN-45), and a normal gastric epithelium cell line (GES-1) were obtained from the Key Laboratory of Environmental Medicine Engineering, The Ministry of Education, Southeast University (Nanjing, China). The cells were cultured in DMEM (GE Health Care HyClone™, Logan, UT, USA) medium supplemented with 10% fetal bovine serum (FBS), 100 U/mL penicillin, and 100 mg/mL streptomycin in humidified air at 37°C with 5% CO_2_.

### RNA isolation and qRT-PCR analyses

Total RNA was isolated from tissues or cultured cells using TRIzol^®^ reagent (Invitrogen, Carlsbad, CA, USA). Using qRT-PCR, the RNA was reverse-transcribed to cDNA by using a Reverse Transcription Kit (Promega, Madison, WI, USA). Real-time PCR was performed to detect the expression of UCA1 with the Step One Plus™ PCR System (Applied Biosystems, Foster City, CA, USA). The qRT-PCR was then performed using the GoTaq^®^ qPCR Master Mix of Power SYBR^®^ Green (Promega) according to the manufacturer's protocol. Results were normalized to the expression of glyceraldehyde 3-phosphate dehydrogenase (GAPDH). PCR primers for UCA1 and GAPDH were as follows: The UCA1 sense primer was 5′-TCCACACCCAAAACAAAA-3′ and the reverse primer was 5′-GCCCTCTAACAACAAACAAC-3′; the GAPDH sense primer was 5′-GGGAGCCAAAAGGGTCATCA-3′ and the reverse primer was 5′- TGATGGCATGGACTGTGGTC-3′. The relative fold-change results were calculated using the 2^-ΔΔCt^ method, where [ΔCt = (Ct _RNAs_ - Ct _GAPDH_) and ΔΔCt = ΔCt _tumor tissues_ - ΔCt _adjacent non-tumor tissues_].

### Association analyses between clinical features and UCA1 expression levels

According to the UCA1 expression in gastric cancer tissue samples, we further analyzed the association between UCA1 and clinical features, including the sex, age, tumor grade, TNM stage, pathological stage, and lymphatic metastases status.

### *In situ* hybridization

RNA *in situ* hybridization was performed according to the manufacturer's protocol (Exon Biotech, Guangzhou, China). The *in situ* hybridization probe (UCA1 gene location, 992–1260) amplification used PCR and cloning of the plasmid containing T3 and T7 promoters. To obtain the UCA1 probe, we used the T3 or T7 reverse transcriptase reverse transcription method, then incorporated the DIG-dUTP to probe the sequence. Briefly, cells were rinsed in phosphate-buffered saline (PBS) and fixed in 4% formaldehyde for 15 minutes at 25°C. The cells were then incubated in mRNA hybridization buffer for 60 minutes at 55°C. The hybridization was performed using the UCA1 probe in a moist chamber at 37–42°C for 16–72 hours according to the established protocol. After RNA *in situ* hybridization, we used an anti-digoxin horse radish peroxidase (HRP)-conjugate and incubated at 37°C for 45 minutes. Subsequently, diaminobenzidine tetrahydrochloride (DAB) and hematoxylin staining were performed. Finally, all images were obtained using a FSX100 (Olympus, Tokyo, Japan) confocal microscope.

### Plasmid construct and transfection of gastric cancer cells

The UCA1 construct was synthesized and integrated into a vector (Abmgood China, Nanjing, China). The upregulated and downregulated expression of UCA1 in cells was accomplished by using the Lenti-CMV-GFP-2A-Puro-UCA1 and the piLenti-UCA1-siRNA-GFP lentivirus vector constructs (10^8^IU/mL), and the empty vector was used as a control. Gastric cells cultured in 6-well plates were transfected with the Lenti-CMV-GFP-2A-Puro-UCA1, piLenti-UCA1-siRNA-GFP, or their corresponding blank control empty vectors according to the manufacturer’s instructions. Puromycin was then used to screen the stably-transfected cell lines. Finally, the expression level of UCA1 was detected using qRT-PCR.

### Cell proliferation and wound healing assays

The proliferation of lentivirus transfected BGC-823 cells was determined using the MTT cell proliferation assay kit (Sigma-Aldrich, St. Louis, MO, USA) according to the manufacturer's instruction. Cells were seeded into 96-well plates (0.3 × 10^5^ cells/well in 100 μL complete medium) and cultured for 12, 24, 48, and 72 hours, then washed with PBS. Thereafter, the medium was replaced by medium (200 μL) containing 0.5 mg/mL MTT, and the mixture was incubated for 4 hours at 37°C and the absorbance was measured at 490 nm using a microplate reader (Bio Tek Instruments Inc. USA).

Lentivirus transfected BGC-823 cell migration was determined using a wound healing assay. The negative control and lentivirus-transfected BGC-823 cells were seeded into six-well plates (5 × 10^5^ cells/well), and after reaching 90% confluency, a scratch was made in the plate with a 200 μL pipette tip. The remaining cells were washed with medium and incubated with 5% CO_2_ at 37°C for 24 hours. The migration distance of cells was photographed using a FSX100 Biological Image system (Olympus) and the images were analyzed using Image Pro^®^ Plus software, version 6.0 (Media Cybernetics, Rockville, MD, USA).

### Invasion assay

Cell invasion was determined using a Transwell^®^ Matrigel^®^ system. Cells (1 × 10^5^ cells/well) were cultured in the top chambers of 24-well Transwell^®^ plates (Corning, Corning, NY, USA) and complete DMEM medium was added to the bottom chambers and incubated with 5% CO_2_ at 37°C for 24 hours. After incubation, the cells on the surface of the top chamber membrane were removed with a cotton swab. Cells that migrated to the bottom surface of the top chamber membranes were stained with 1 mg/mL Crystal Violet and hematoxylin solution, and the stained cells were counted cells using a FSX100 microscope (Olympus). The experiments were performed in triplicate and repeated.

### Flow cytometry assay

Cells were cultured in six-well plates to 80% confluency and harvested using trypsin (0.25%), and then washed twice with PBS. Quantitation of cell apoptosis used the Annexin V-APC 7AAD apoptosis kit (MultiSciences Biotech, Hangzhou, China) according to the manufacturer’s instructions. A flow cytometer (Becton Dickinson Medical Devices Co Ltd., Franklin Lakes, NJ, USA) was used for detection and analyses.

### Western blot assay and antibodies

Cells were collected for the preparation of a cell lysate, which was separated using SDS-polyacrylamide gel electrophoresis (SDS-PAGE), then transferred to a nitrocellulose membrane as previously described [46, 47]. The membrane was blocked using 5% milk in tris-buffered saline Polysorbate 20 (TBST) for 2 hours, then incubated with primary antibodies (1:1000) in 5% bovine serum albumen (BSA)-TBST at 4°C overnight. Finally, the membrane was washed using TBST and incubated with corresponding secondary antibodies for 1 hour. The ECL chromogenic kit (Thermo Fisher, Scotts Valley, CA, USA) was used to detect the protein bands. The images were captured using a Tanon-5200 chemiluminescent imaging system (Tanon Science & Technology, Shanghai, China). Antibodies were purchased from Cell Signaling Technology (Danvers, MA, USA).

### Tumor formation analyses using a nude mouse model

Male athymic BALB/c nude mice were fed for 4 weeks in pathogen-free conditions. The tumor cell injection protocols were approved by the Committee on the Ethics of Animal Experiments of the Southeast University. BGC-823 cells were transfected with lentivirus and harvested from cell culture plates, washed with PBS, and resuspended at a concentration of 5 × 10^6^ cells/200 μL, and subcutaneously injected into the dorsal flank of each nude mice. The growth of tumors was examined every 5 days, and the tumor volumes were calculated using the formula: V= (πab^2^)/6, (V, volume; a, longitudinal diameter; b, latitudinal diameter) [48]. Twenty days later, the mice were sacrificed and the tumors were dissected and weighted. We also detected related key protein expressions of UCA1 and key components of the signal pathway by qRT-PCR and immunohistochemical analyses. This study was performed in accordance with the recommendations of the Guide for the Care and Use of Laboratory Animals of the National Institutes of Health.

### Statistical analysis

Quantitative data were expressed as the mean ± standard deviation. Statistical analyses were performed using SPSS statistical software for Windows, version 20.0 (SPSS, Chicago, IL, USA). Statistical significance was determined using the Student’s *t*-test or the chi-square test as appropriate. P values < 0.05 were considered significant.
